# Treatment of Third Molars

**Published:** 1886-01

**Authors:** A. Berry


### Treatment of Third Molars.

BY A. BERRY, D.D.S.

Read before the Ohio State Dental Society.

Mr. President and Gentlemen of the Ohio State Dental Society:—The question of the proper treatment of impacted third molars, is often of grave importance. A patient complains of trouble in the region of an inferior third molar, often slightly elevated above the alveolus, nearly or quite and covered by the gum, which is highly inflamed, exceedingly sensitive and painful.

In probably a majority of such cases, when the annoyance is recent from a third molar being erupted at about the usual period, and firmly imbedded, indicating difficulty in its removal, if section of the gums and local treatment fail, it is well to extract the second molar, when the swelling and pain will usually soon cease, and the third molar take the place of the second.

But when the trouble has been of long standing, rendering it probable that the advance of the third molar has been permanently stopped, the necessity of its removal is clearly indicated. Now, serious fears may confront the operator. Insuperable difficulty, or want of fortitude in the patient, may cause failure in an effort to extract the tooth. Vivid recollections of trouble in similar instances, as well as the possible herculean task, present themselves in fearful array to the mind of the operator. He recollects that an able dentist of long and large experience, after great annoyance in the matter, was mulcted by verdict of a jury of several thousands of dollars, as the result of an attempt to extract a third inferior molar under exceedingly difficult conditions.

But he has no way of escape. He cannot, like the neighborhood tooth-puller, say to the patient: "The roots of this tooth are hooked under the jaw bone, and it cannot be pulled without breaking the jaw." He belongs to the Æsculapian family. His work is to prevent or alleviate human suffering; and he must do something to afford relief to his patient. He must remove the tooth or make a failure. He prepares the patient for the ordeal as he thinks best, telling him, perhaps, that the roots of the tooth may be crooked, causing difficulty in elevating it, and that with a poor chance to apply force, the operation may be tedious as well as painful; but encourages him with the prospect of relief.

With his physick, hawks-bill, and several small beaked forceps and elevators of various forms and sizes, and an anæsthetic for local application, the dentist is ready for action. After careful examination to decide as to the best manner of applying force, he commences with, perhaps, a small elevator deeply inserted between the second and third molars, and makes an effort to loosen and raise the offending tooth. If he succeeds and cangrasp it with forceps and extract it, even after prolonged effort, he has reason for congratulating his patient as well as himself. But in some cases if he attains this result after a half hour or more of presistent effort, causing intense suffering, he may consider it a fortunate ending of the dreaded ordeal.

After giving suitable directions, and charging a fee considerably larger than for ordinary tooth-drawing, he dismisses the patient. For all this severe mental and physical effort, the principal reward of the dentist is the consciousness of having done his duty. But his trouble does not always end here. Severe inflammation and pain may follow, requiring further treatment, and convalescence may be so tardy as not to be agreeable to the operator nor satisfactory to the patient.

Most persons, fortunately for them, suppose that all teeth can be extracted readily; not knowing the difficulties in some instances with third inferior molars. As every one considers himself a competent judge, the dentist, in cases like this, may bless his stars if the patient and all his friends do not make the neighborhood lively with tales of his awkwardness in being so tedious and causing so severe suffering in extracting a tooth.

